# Autworks: a cross-disease network biology application for Autism and related disorders

**DOI:** 10.1186/1755-8794-5-56

**Published:** 2012-11-28

**Authors:** Tristan H Nelson, Jae-Yoon Jung, Todd F DeLuca, Byron K Hinebaugh, Kristian Che St Gabriel, Dennis P Wall

**Affiliations:** 1 The Center for Biomedical Informatics, Harvard Medical School, Boston, MA 02115, USA; 2 Department of Pathology, Beth Israel Deaconess Medical Center, Boston, MA 02115, USA

**Keywords:** Autism, Autistic disorder, Autism spectrum disorders, Autism genetics, Autism genomics, Network biology, Network medicine, Translational bioinformatics, Protein-protein interactions

## Abstract

**Background:**

The genetic etiology of autism is heterogeneous. Multiple disorders share genotypic and phenotypic traits with autism. Network based cross-disorder analysis can aid in the understanding and characterization of the molecular pathology of autism, but there are few tools that enable us to conduct cross-disorder analysis and to visualize the results.

**Description:**

We have designed Autworks as a web portal to bring together gene interaction and gene-disease association data on autism to enable network construction, visualization, network comparisons with numerous other related neurological conditions and disorders. Users may examine the structure of gene interactions within a set of disorder-associated genes, compare networks of disorder/disease genes with those of other disorders/diseases, and upload their own sets for comparative analysis.

**Conclusions:**

Autworks is a web application that provides an easy-to-use resource for researchers of varied backgrounds to analyze the autism gene network structure within and between disorders.

Availability: http://autworks.hms.harvard.edu/

## Background

Autism spectrum disorder (ASD) is one of the most common [1,2] and highly heritable [3,4] neurodevelopmental disorders. Despite, and perhaps because of, its commonality, ASD is known to have a highly complex genetic etiology [5-8].

More than 100 genes have been reported to be perturbed in individuals with ASD [9], while only a few such genes have been verified by genome-wide association studies or gene expression analyses. There are a number of Mendelian disorders in which patients may manifest ASD traits, including fragile X syndrome [10], tuberous sclerosis complex [11], Rett syndrome [12], and Angelman syndrome [13] to name a few. Finally, common copy number variations have been found between ASD and other complex mental disorders, including schizophrenia (e.g., 1q21.1 duplication), bipolar disorder (e.g., 16p11.2 duplication), and epilepsy (e.g., 17q12 deletion) [9,14].

The complexity of the autism genetic landscape suggests that networks of candidate genes will be of value to identify biological themes that contribute to the spectrum of autism phenotypes. Innovative work by Rzhetsky et al. [15] demonstrated that significant correlations of comorbidity were found across a wide range of disorders, ASD in particular. Related research demonstrated that combining the known genetic etiology of behaviorally related disorders with ASD through biological networks could provide particular insight into genes with great significance in ASD, as well as predictive power for novel autism-associated variants [16].

To capitalize on the value of network biology and a cross-disease understanding of autism, we built a web application called Autworks to allow for network scale cross-disorder analysis. We aimed to integrate pathogenic and genomic information spread across different data repositories and to augment this with novel features including network analysis and visualization tools lacking in existing applications. To this end, Autworks incorporates disease-specific, manually curated databases that provide gene associations for a few major mental disorders (e.g., [17-20]), pathogenic information from general-purpose genetic databases, including GeneCards [21], HuGE Navigator [22], and PharnGKB [23], as well as our own PubMed-based gene-disorder search results. Finally, we examined gene/protein interaction databases [24-28] and incorporated their functionality into Autworks, as these repositories mainly focus on general cellular interactions without disease-context.

## Construction and content

### Genes and disorders

Genes constitute nodes in our network analysis. 19,191 protein-coding human genes and their descriptive data are derived from the official list of human genes provided by the HUGO Gene Nomenclature Committee (HGNC) [29]. Diseases are derived from the 2012 version of Medical Subject Headings (MeSH). Specifically, disorder names and their entry terms were gathered for all terms in sub-tree categories from C04 (Neoplasms) to C20 (Immune System Diseases) as well as F03 (Mental Disorders), in order to capture disorders with a possible genetic etiology.

### Gene-disorder associations

For each disorder, gene-given disorder associations are derived from a PubMed search linking the given disorder name with gene names. Disorder names were derived as described above. Each MeSH term as well as all associated entry terms was queried for gene associations--the results were combined for a list of all gene-disorder associations. This produces 2,711 disorders with 660,090 associations with 16,685 genes.

### Gene-gene interactions

Gene interaction network data is based on the Search Tool for Retrieval of Interacting Genes/Proteins (STRING) [26]. In Autworks, the evidence for a gene interaction is broken down along five different lines of evidence, including protein-protein interactions, pathway information, and co-expressed arrays. Networks can be constructed using specific lines of evidence, omitting evidence not considered useful for a particular inquiry. By default, interactions with a medium score as defined by STRING (400 or greater) constitute valid interactions. Ensembl [30] was used to map proteins from STRING to genes in Autworks. This produces 367,308 interactions between genes in Autworks.

### Disorder-disorder associations

Autworks contains a measure of association between disorders based on the hypergeometric distribution. By considering the common genes shared by two disorders, this method tests the likelihood of seeing this amount of overlap (or more) by random chance. Enrichment values were calculated using the GNU Scientific Library. For each pair of disease gene sets [D1;D2] and the set of all human protein-coding genes associated with any disorder [G] we calculated the disease enrichment score as a p-value by applying the function gsl_cdf_hypergeometric Q(k; n1; n2; t) with the following parameters: k = |D1 ∩ D2| - 1 , n1 = |D2|, n2 = |G|, t = |D1|. Sets containing fewer than 5 genes were not considered for this analysis.

## Utility

**Figure 1:**
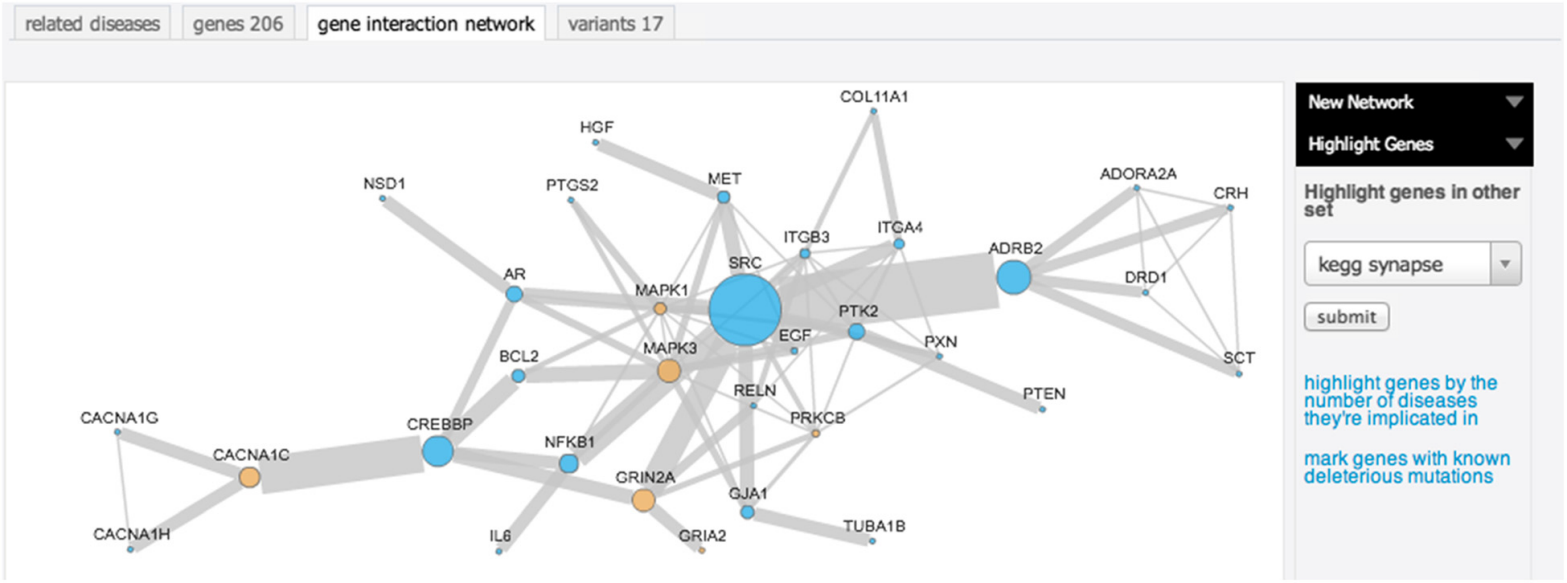
**The network of autism candidate genes.** Through Autworks this network is searchable and tunable with parameters that alter the visibility of gene-to-gene interactions.

For each disease Autworks provides four distinct views: a cross-disorder view that visualizes a network of disorders against which the current disorder has significant genetic overlap, a list view of genes in the set ordered by significance, a gene network view that interactively shows the relationship of genes within a given disorder, as shown in Figure 1, and a genetic variant view listing known variants associated with the disease via genome wide association studies.

The gene network visualization component of Autworks contains several features that allow researchers to use only the genes and interactions they consider interesting. Researchers can choose to display interactions based on pathway databases, experimentally proven interactions, co-expression, or some combination of the above. Details of the data behind each interaction are linked to from the network visualization. In addition to the evidence behind the interaction, this view shows any diseases the two genes have in common, as well as places in the literature in which they occur together. Autworks also allows researchers to highlight genes that belong to other sets, are implicated in a large number of other disorders. For each interaction, specific information about the type of interaction as well as papers that mention both genes together are available.

## Discussion

As an demonstration of a single gene investigation in Autworks, here we show a Mendelian syndrome which shares phenotypic traits with autism spectrum disorder. Rubinstein-Taybi syndrome can be caused by a mutation in the gene which codes for CREB binding protein (CREBBP [31]). The main symptoms of this disorder include slow development of cognitive and motor skills, and researchers have speculated about classifying the syndrome among the autisms [32]. A search for this gene in Autworks reveals that this gene occupies a place in the largest network of genes implicated in autism, mediating inter-actions between genes responsible for the calcium channel receptors (CACNA1C, CACNA1G, CACNA1H), mutations in which are responsible for Timothy syndrome, another syndromic variant of autism [33], and a larger network of genes associated with autism through genome wide association studies (GWAS) and gene expression experiments (AR, BCL2, GRIN2A, NFKB1, among others). These interactions are based on annotated gene pathways, including the MAPK signaling pathways [34] and the JAK-STAT signaling pathways [35]. The position of this gene in the autism interaction network and the association of this gene with a disorder that has an autism-like phenotype make it an interesting candidate for further inquiry. This gene interaction based evidence for the association of CREBBP with autism genes is not known to have been reported in the literature before. Autworks makes this type of inquiry remarkably easy to perform.

Researchers may also approach the site with interest in a set of genes, possibly ones derived from one's own experiments, experiments reported in the literature, or biological processes. As a particular example of this, researchers have expressed interest in disruption of the glutamatergic synapse as a possible causative factor of autism. The pathway is well described in the Kyoto Encyclopedia of Genes and Genomes (KEGG), with a list of genes and interactions involved in the pathway. Importing the set of genes from this pathway into Autworks provides an enrichment analysis against the set of disorders in Autworks. As this set describes a neurologic function, it is significantly enriched for many different neurological disorders, including autism. To get a sense of how this relates to the network of interactions in autism, one can mark the genes occurring in this set on the autism network using Autworks as shown in Figure 1. One can clearly see a significant number of the genes in this pathway in the largest network of interacting autism genes, suggesting that this pathway may indeed play a significant role in autism.

## Conclusions

Autworks provides a dynamic picture of the network of autism candidate genes and a set of network biology methods for researchers of varied backgrounds. These methods should assist in the analysis of the autism network and its relationship with the networks of other, related human disorders and in advancements towards a clearer understanding of the genetic landscape of autism.

## Availability and requirements

Project name: autworks

 Project home page: http://autworks.hms.harvard.edu

 Operating system(s): platform independent

 Programming Language: Ruby

 Other requirements: HTML5 compliant web browser

## Competing interests

The authors declare they have no competing interests.

## Authors’ contributions

DPW conceived the study and participated in application evaluation and design. THN and JYJ developed the application and wrote the manuscript and participated in application evaluation and design. TFD edited the manuscript and participated in application evaluation and design. All authors read and approved the final document.

## Pre-publication history

The pre-publication history for this paper can be accessed here:

http://www.biomedcentral.com/1755-8794/5/56/prepub
